# The potential of aerosol eDNA sampling for the characterisation of commercial seed lots

**DOI:** 10.1371/journal.pone.0201617

**Published:** 2018-08-01

**Authors:** Lorretha C. Emenyeonu, Adam E. Croxford, Mike J. Wilkinson

**Affiliations:** 1 School of Agriculture, Food and Wine, Waite Campus, Urrbrae, The University of Adelaide, Adelaide, SA, Australia; 2 Pwllpeiran Upland Research Centre, Institute of Biological, Environmental & Rural Sciences, Aberystwyth University, Penglais, Aberystwyth, Ceredigion, United Kingdom; University of Helsinki, FINLAND

## Abstract

Seed shipments, silos and storage houses often contain weed seeds or seeds of restricted crops such as undeclared genetically modified (GM) varieties. Random sub-sampling is the favoured approach to detect unwanted biological materials in seed lots but is prohibitively expensive or else ineffective for the huge volumes of seeds moved in commercial operations. This study uses maize and cowpea seed admixtures as an exemplar to evaluate the feasibility of using aerosol sampling of “seed dust” as an alternative to seed sub-sampling. In an initial calibration phase, qPCR of the *rbcL* barcode followed by high-resolution melting (HRM) of a DNA titration series revealed a strong linear relationship between mix composition and HRM profiles. However, the relationship became skewed when flour mixes were used to build the titration, implying a DNA extraction bias favouring cowpea. Aerosol samples of seed dust above a titration of mixed seed samples were then collected along vertical and lateral axes. Aerosols were characterised by light microscopy, qPCR-HRM and next-generation DNA sequencing (Illumina MiSeq). Both molecular approaches again showed bias but this time in a reverse direction to flour samples. Microscopic examination of the aerosol sample suggested this divergence could be attributed to differences in abundance of airborne starch particles. Despite the bias, it was nevertheless possible to estimate relative abundance of each species using the abundance of minibarcodes. In light of these results we explore the feasibility of aerosol sampling for commercial seed lot characterisation.

## Introduction

Most countries impose import restrictions to control the accidental or intentional introduction of seeds from unauthorised crops, invasive and noxious weeds, or more recently, from genetically modified (GM) seeds [1, 2]. Enforcement of these restrictions is made difficult by the sheer volume of seed involved in commercial operations and by the complexity of seed handling processes. It is therefore unsurprising that there have been regular reports of unwanted admixtures of seed lots throughout the world [3–6]. Since not all types and levels of admixtures are a cause for concern, it is important that the legislature specifies which seeds are prohibited [3, 7] and sets acceptable thresholds for the presence of contaminants. These thresholds vary between jurisdictions and contexts. For example, in the United States (US) a seed lot may contain up to 2% by weight of seeds for most weeds but there is zero tolerance for noxious weeds [2]. In comparison, Nigeria imposes a blanket of 0.05% by weight limit for the presence of any weed seeds [8]. There is a similar range of tolerance for the adventitious presence of undeclared GM seeds, with the European Union requiring less than 0.9% GM presence, but the US and Canada allowing up to 5% [9]. Enforcement of these thresholds on a commercial scale is reliant upon an adequate testing regime. However, the ability of any diagnostic protocol to quantify seed admixtures is overwhelmingly influenced by the sampling approach used to isolate seeds for testing [10, 11]. Indeed, sampling errors due to non-random distribution of seed lot contaminants are estimated to be 10–100 times greater than errors due to the analysis method chosen [12].

Random sub-sampling of seeds is the current preferred method of choice to characterise the presence of unwanted seeds within seed lots but is still faced with a number of challenges. For instance, the distribution of unwanted seeds within seed lots is typically heterogeneous [13] and so necessitates the use of an appropriate sampling protocol to ensure an adequate spread of sampling points to accommodate for uneven presence of contaminant seeds. This challenge is made more difficult by the huge volumes of seeds involved in commercial operations and by logistical constraints that can dictate when and where sampling can occur. Cost is also an important variable. On the one hand, the more seed samples that are tested, the more representative the estimate of admixture will become. On the other, increasing sample size increases the costs of sampling and can severely delay commercial operations. There is also a hidden additional cost for nations adopting low admixture tolerances since these thresholds require much larger test sample sizes to provide statistical confidence of contamination. In the light of these considerations it is perhaps unsurprising that many alternative protocols for seed sampling have been developed by international and local organisations e.g. [14, 15] and that there is a notable lack of consensus on seed sampling protocols [12].

Sampling protocols are developed and adopted on the basis of both statistical and non-statistical factors. The statistical factors include threshold of uncertainty and randomness of the lot [16] while the non-statistical considerations include time and cost of performing the tests [17]. The presence of non-statistical considerations in protocol selection is important because it impedes progress towards global harmonisation of seed testing practices. Viewed in this context, there is a strong need for new seed testing strategies that significantly reduce financial and time costs so that statistical considerations alone can drive protocol selection.

All seed handling facilities generate aerosols of particulates from seed fragments and other plant, pest and fungal sources [18]. Airborne particles of this kind range in size from 1nm to 100μm in size [19]. The composition of aerosols is ultimately determined by the size and proximity of source materials and this property can be exploited to characterise features of the source [20, 21]. There are numerous studies of this kind for pollen aerosols [22–24] which include some that have used modelling approaches for the reconstruction of the location and size of pollen sources on a landscape scale [25, 26]. These developments raise the intriguing possibility of developing aerosol-based strategies to characterise commercial seed lots. To date, characterisation of aerosols of seed handling facilities has largely focussed on issues associated with workplace health [27]. To our knowledge, there have been no attempts to use an aerosol sampling approach to characterise the seed lot itself. The aim of this study is therefore to evaluate the long-term feasibility of developing an aerosol-based protocol to characterise admixtures in seed lots. For this purpose we use admixtures of cowpea (*Vigna unguiculata*) and maize (*Zea mays*) seeds as exemplar source materials.

## Materials and methods

### Sample preparation, DNA extraction and standardisation

This study used dried maize and cowpea grains obtained from commercial outlets (maize from Afrikan Continental, 391B Prospect Rd, Blair Athol SA 5084, Australia Lat:-34.8596168 Lon: 138.59248680; cowpea from Adelaide Central Market, 44–60 Gouger St., Adelaide SA5000, Australia Lat: -34.9295219 Lon: 138.572832) to best represent the condition of grains in the supply chain. DNA was extracted from the ground seeds (flour), from seed mixes (based on weight) and aerosol samples using ISOLATE II Plant DNA Kit (Bioline, Australia) following the manufacturer’s instructions and quantified using a NanoDrop spectrophotometer (Thermo Fisher Scientific, USA).

#### DNA titration series

The DNA extracted from pure maize and cowpea flour was further assessed by qPCR on a Rotor-Gene 6000 cycler (Corbett Research, Australia) and concentrations were standardised to 20ng/μl. The following titration series of DNA mixtures (cowpea DNA in maize DNA) were then created in replicates of five: 0%, 10%, 20%, 30%, 40%, 50%, 60%, 70%, 80%, 90% and 100%.

#### Flour mix titration series

The flour mix titration series was created by combining maize and cowpea seeds in different weight proportions (0%, 5%, 10%, 30%, 50%, 70%, 90%, 95% and 100%) which were ground into fine powder using a mortar and pestle. DNA was then isolated from 20mg of each powder sample using the ISOLATE II Plant DNA Kit (Bioline, Australia). There were three replicates for each point in the titration series.

#### Aerosol calibration and titration series

Plastic bins (surface area = 840cm^2^, depth = 17.5cm) were filled with 10kg of cowpea and /or maize grains in varying proportions to create a titration series. All aerosol experiments were performed in a draft-proof, windowless room approximately 3m × 3m × 2m in size. A 3-5h interval was left between removal of the seed bin (source) after one session of aerosol sampling and the introduction of another seed bin. This allowed time for airborne particles to sediment between sampling times. The use of small (10kg) seed bins as the source of the aerosols was intended to minimise aerosol carry-over between sampling times. Each bin of the seed titration series was mixed by weight to a final concentration of 0%, 5%, 10%, 20%, 30%, 40%, 50%, 60%, 70%, 80%, 90%, 95% and 100% (cowpea in maize grains). Aerosol samples were collected from the seed mixes in two ways: by using a purpose-built volumetric spore trap, the Coriolis μ aerosol sampler (Bertin Technologies, France); and by impaction on muslin cloth (100% cotton) for two minutes placed over the entry port of a Philips Handheld Vacuum (Model FC6140) set at an airflow rate of 620 l/min. There were three replicate samples collected for each point in the titration series. The Coriolis sampler pulled air into a cone pre-filled with 1ml of water at an airflow rate of 300 l/min for 2 min [28, 29]. The aspirated particles were separated from the airflow by centrifugal force and aggregated against the wall of the cone. The liquid was spun again to concentrate the particles at the bottom of the cone. Five replicates of air samples were also collected above bins containing maize or cowpea grains at the following heights: 5cm, 10cm, 15cm, 30cm, 60cm and 120cm, and also along a lateral transect (5cm, 10cm and 15cm) from the bin edge at a height of 5cm above the bin. The Coriolis samples were collected from seed mixes of the titration series at a height of 60cm directly above the bins and the vacuum samples were collected from the same titration series at the height of 10cm.

### Physical characterisation of maize and cowpea aerosols

Microscopic analysis was conducted on the aerosol samples obtained from vertical and lateral transects from maize and cowpea seed bins described above to determine the effect of sampling position on airborne particle profiles. To remove extraneous particles, Teflon well slides and coverslips were washed in a mixture of RO water and Quantum Clean detergent (Rowe Scientific, Australia), rinsed in RO water and ethanol and allowed to air-dry. The sample particles were re-suspended in 10μl of water and 1μl of Lugol’s iodine [30] was added to 1μl of each sample (to differentiate between starch and non-starch particles) placed on a Teflon well slide. Two negative controls were prepared using 1μl of Lugol’s iodine added to 1μl of water and placed on a dry Teflon slide. All the samples were analysed using a Nikon Eclipse (Ni-E) microscope with NIS-Elements Advanced Research (AR) imaging software (Nikon, USA). All images were captured as Red Green Blue (RGB) 8bit images using the 10x objective and 10x Differential Interference Contrast (DIC) to give the particles a 3-Dimensional shape. The images were analysed using the ImageJ Software (National Institute of Health, USA) (http://imagej.nih.gov/ij/).

### PCR primer design

We first sought to design a set of primers that universally matched both target species but which also contained internal sequence variation that could be exploited for species diagnosis. For this we retrieved the *rbcL* gene sequences of the two species from the National Center for Biotechnology Information (NCBI) online database, and aligned using the MultAlin online software. A pair of internal PCR primers were designed within the region targeted by the universal *rbcL* barcoding primers [31–34] using Primer3 software (http://bioinfo.ut.ee/primer3/). The internal primers (forward 5’ GATACTGATATCTTGGCAGC 3’ and reverse 5’ GTTGTCCATGTACCAGTAG 3’) were designed to be an exact match for both maize and cowpea, thereby minimising the potential for amplification bias. These primers were further assessed using the NetPrimer online software (http://www.premierbiosoft.com/NetPrimer/AnalyzePrimer.jsp) to ensure optimal site selection for PCR amplification. The predicted amplicons contain 10 SNPs between the two species in an expected length of 107bp which is within the optimal size threshold for HRM [35, 36].

### Species quantification by qPCR

We performed qPCR using the Rotor-Gene 6000 platform (Corbett Research, Australia) to determine the amount of PCR-amplifiable DNA isolated from the pure aerosol samples at the assigned collection heights and distances. Each 10μl reaction mixture contained 2μl of DNA extract, KAPA SYBR FAST qPCR universal master mix (Kapa Biosystems, South Africa) and 0.1μM of forward and reverse internal *rbcL* primers. The qPCR protocol included initial denaturation at 95^°^C for 10 min, followed by 35 cycles of denaturation at 95^°^C for 5s, annealing at 58^°^C for 30s, and extension at 72^°^C for 30s. Serial dilutions (1/10, 1/100, 1/1000, 1/10000, 1/100000) of pure DNA extracted from maize and cowpea grains were used as standards. Correlation analysis between the DNA yield and particle size and abundance profiles (described above), was performed using XLSTAT Version 2015.5.01.22724 (Addinsoft, USA).

### PCR for High Resolution Melting (HRM) analysis

The PCRs for HRM analysis were performed in 10μl volumes containing 2μl of DNA extract; SensiFAST HRM mix (Bioline, Australia) and 1.0μM forward and reverse internal *rbcL* primers. PCR amplification was conducted on the Rotor-Gene 6000 cycler (Corbett Research, Australia) using the following protocol: initial denaturation at 95^°^C for 10 min, followed by 35 cycles of denaturation at 95^°^C for 5s, annealing at 58^°^C for 30s, and extension at 72^°^C for 30s.

### High Resolution Melting analysis

HRM was performed on three categories of admixtures: seed DNA mixtures (DNA extracted from each seed source separately and mixed), DNA extracted from flour mixtures (seed titration mixtures based on weight) and eDNA from aerosol samples collected above 10kg boxes of mixed seeds (seed mixture compositions based on weight). HRM was conducted on the Rotor-Gene 6000 machine (Corbett Research, Australia) using a temperature ramp from 75–90^°^C in incremental steps of 0.1^°^C every 2s and DNA melt curve analysis was performed using the Rotor-Gene 6000 Series Software Version 1.7.87 (Corbett Research, Australia). The melt data for the normalised fluorescence curves was exported and analysed using GraphPad Prism Software Version 6.07 for Windows.

### Illumina MiSeq sequencing

#### PCR amplification and library construction

Illumina sequencing was performed on amplicons from the seed flour and aerosol admixture samples. A modified two-step PCR amplification approach [37] was used to generate tagged PCR amplicons using primer pairs with unique indices. In the first PCR step, PCR amplification of the barcode region of *rbcL* gene was performed by adapting the method described by Giraud [38]. The amplification was performed on a BIO-RAD T100 Thermal Cycler (BIO-RAD Laboratories, Inc., Singapore) in 20μl reactions containing 20-40ng of DNA, 1.6U MyFi DNA polymerase (Bioline, Australia), MyFi reaction buffer (Bioline, Australia) and 4μM of each primer. Two sets of Illumina-modified *rbcL* primers were designed and used in the MiSeq run to create cluster diversity on the flow cell: *rbcL* forward primer/P5 and reverse primer/P7; and forward primer/P7 and reverse primer/P5. The amplification conditions consisted of an initial denaturation step at 95^°^C for 1min, followed by 35 cycles of 95^°^C for 15s, 55^°^C for 15s and 72^°^C for 15s, followed by a final extension at 72^°^C for 5min. Amplicons were then purified using Agencourt AMpure XP beads (Beckman Coulter, USA) following the manufacturer’s instructions.

The second PCR was performed to add indices and complete the adapters on the amplicons from the first PCR step using the Illumina Nextera adapter kit (Illumina, USA). Here, 12.5μl reaction mixtures were used that contained 2.5μl of the purified PCR product, MyFi reaction buffer (Bioline, Australia), 1U MyFi DNA polymerase (Bioline, Australia.) and 1.0μM of each forward and reverse indexed primers. The PCR conditions consisted of an initial denaturation step at 95^°^C for 1 min, followed by 5 cycles of 95^°^C for 15s, 55^°^C for 15s and 72^°^C for 15s. A second purification was then conducted using Agencourt AMpure XP beads (Beckman Coulter, USA) as described above.

#### Multiple bulks to normalise variation between samples

We adopted a multiple pooling strategy to moderate the influence of outlier samples. Following purification, samples were combined into 16 bulks, each containing eight individual samples. Following a 1:50 dilution of each sample, a qPCR assessment was performed by adding 1μl purified indexed amplicons into the KAPA SYBR FAST qPCR universal master mix (Kapa Biosystems, South Africa) 1.0μM forward and reverse Illumina sequencing primers (Illumina, USA) in a total of 10μL reaction volume. Serial dilutions of 10μM PhiX of 1/2, 1/10, 1/100, 1/1000, and 1/10000 were used as standards for quantification. The qPCR conditions included an initial denaturation step of 95^°^C for 10min, followed by 30 cycles of 95^°^C for 5s and 60^°^C for 30s. DNA concentrations were calculated using the Rotorgene 6000 software (Corbett Research, Australia) and the concentrations of all 16 bulks were standardised to create one large bulk of samples at 2.4nM concentration. This was further diluted to 12pM and sequenced using the Illumina 600-cylce Version 3 reagent kit (Illumina, USA) by paired-end, 300 cycles on an Illumina MiSeq sequencer at the University of Adelaide.

#### Data analysis

Sequence data was processed using the MiSeq Reporter software (Illumina, USA) to remove poor quality and incomplete reads and to group sequences based on their unique indexing. FASTQ files of *rbcL* sequences passing these filters were then subjected to nBLAST searches (default settings using Notepad++) against at least two alternate pairs of 10bp mini-barcode sequence motifs that differed between the two species, viz: GTTGG**A**TT**T**A (maize) versus GTTGG**G**TT**C**A (cowpea); **C**TTA**C**TA**C**AC (maize) versus **A**TTA**T**TA**T**AC (cowpea); **G**TAATT**T**TT**T** (maize), **A**TAATT**C**TT**A** (cowpea) and/or CATAA**CAC**GC (maize) versus CATAA**ACA**GC (cowpea). Estimates of the relative species abundance in a sample were near-identical regardless of the mini barcode pairing used. For this reason, a simple arithmetic mean (of minibarcode pairings estimates) was used to calculate the proportion of species in each sample.

## Results

### Construction of a calibration curve from the DNA titration series

**Fig 1:**
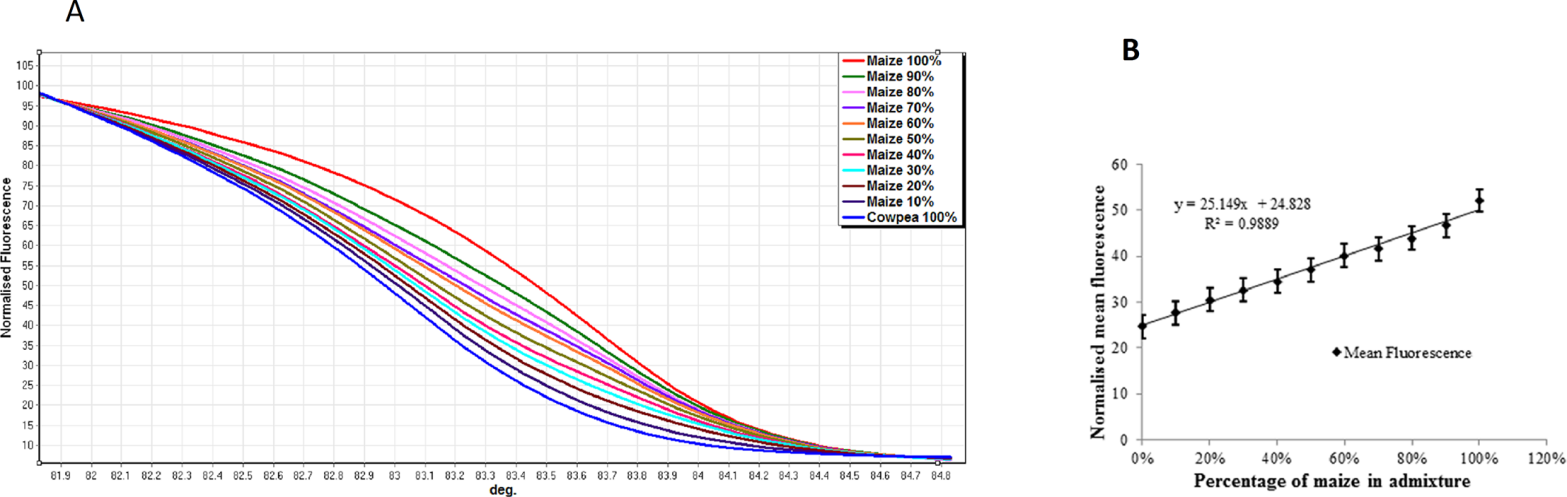
HRM curve profiles from maize and cowpea DNA titration. (**A)** Normalised High Resolution Melting (HRM) curve profiles of *rbcL* amplicons generated from mixtures of maize DNA and cowpea DNA according to the titration series: 0, 10, 20, 30, 40, 50, 60, 70, 80, 90, and 100% maize DNA (in cowpea DNA). Melt curve shapes reflect differences in the sample composition. (**B)** Relationship between HRM fluorescence and maize DNA concentration. Mean fluorescence values recovered from normalised High Resolution Melting (HRM) profiles of maize DNA in maize + cowpea DNA mixtures at 83.5^°^C. The graph shows a strong linear relationship between mean fluorescence and the species composition in the DNA admixture. Error bars represent standard error of the mean.

There was a chronological, stepwise and progressive change in the HRM curves that represented the template species mixes in the DNA titration series (Fig 1A). Pure cowpea samples had the lowest melting temperature, followed in order of increasing content of maize DNA within the template mix and with the pure maize samples melting last. There was also a marked difference in the melt curve shapes of the two pure samples, with those of cowpea (Fig 1A blue line) decaying markedly more rapidly than pure maize (Fig 1A, red line). Melt curve shapes of intermediates in the titration series also reflected the species composition in the template mixture (Fig 1A). Linear regression analysis of the normalised fluorescence at 83.4^°^C showed a strong linear relationship between the concentration of DNA template and fluorescence of the melt, with an R^2^ value of 0.9889 (Fig 1B).

## Accommodating for differential efficiency in DNA extraction

**Fig 2:**
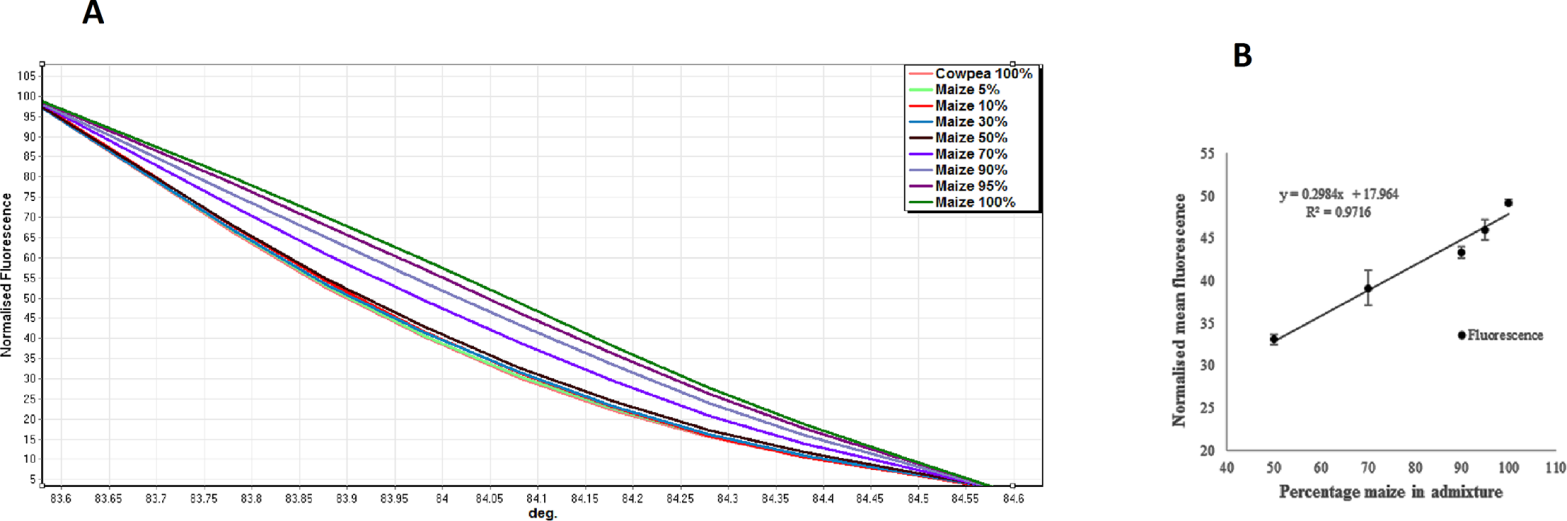
HRM curve profiles from a titration of maize + cowpea flour mixtures. Normalised High Resolution Melting (HRM) analysis of *rbcL* amplicons generated from template DNA extractions from ground flour of maize and cowpea grain according to the titration series: 0, 5, 10, 30, 50, 70, 90, 95 and 100% maize grains (in cowpea grains). **(A)** Normalised HRM curves of DNA from each of the ground seed mixtures. (**B)** Linear regression for HRM profile of cowpea and maize seed flour mixes at a melt temperature of 84.1^°^C. Error bars represent the standard error of the mean. Linear regression of samples containing >50% maize can be described by the relationship y = 0.2984x + 17.964 and has an R^2^ value of 0.9716.

Having established that HRM melt curve analysis could be used to describe a DNA template titration series, the next step was to establish whether the same approach could be used to reflect mixtures of flour samples made from ground grains of the two species. This is a necessary step as any differences in the efficiency of DNA extraction from grains of the two species would introduce divergence from the simple linear relationship observed above between DNA species mix and HRM profile. In the event, there was clear divergence between the relationship revealed from the flour titration series (Fig 2A) and that of the DNA titration series (Fig 1). Whilst there was again good separation between pure samples of the two species (Fig 2A), with cowpea (pink line) decaying markedly more rapidly than pure maize (green line), separation of admixtures was only possible for sample mixes containing >50% maize. Above this threshold, however, admixtures separated well and conserved the order of template species composition. The limit of detection of any maize admixture in cowpea flour samples by HRM was thus deemed to be around 50%, above which a linear relationship was retained as in the DNA mix series (Fig 2B, R^2^ = 0.9716; Fig 1B).

### Physical characterisation of aerosol samples

#### Effect of sampling position on airborne particle profile

**Fig 3:**
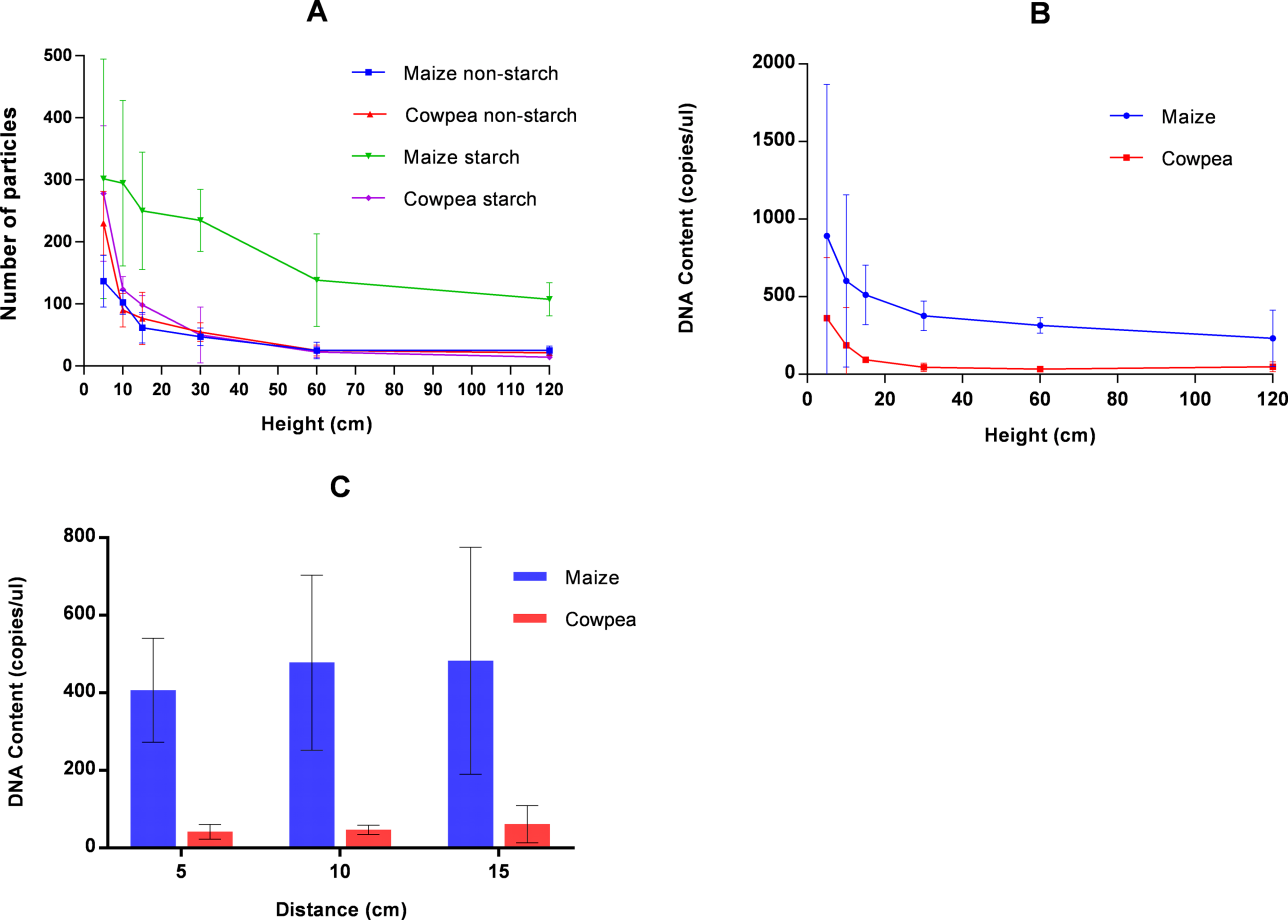
Relationship between aerosol particle profile and sampling position as well as DNA isolated from pure maize and cowpea. (**A)** Decay of airborne tissue (non-starch) and starch particles at various heights above bins containing maize and cowpea grains. Error bars represent 2x standard deviation. (**B)** Concentration of maize and cowpea DNA recovered from aerosol samples collected at different sampling heights (H) in cm from grain bins containing maize or cowpea grains. Error bars indicate 2x standard deviation and account for 95% of variation. (**C)** Concentration of maize and cowpea DNA recovered from aerosol samples collected at different sampling distances (in cm) from grain bins containing maize or cowpea grains. Error bars indicate 2x standard deviation and account for 95% of variation.

Two basic particle types were observed from aerosol samples of both species: starch particles and tissue fragments of various sizes (i.e. non-starch particles). Airborne tissue fragments of each species were present at near identical densities at a height of 60cm (Fig 3A). However, when comparing the starch densities at the same height, there were markedly more maize starch particles than those of cowpea (Fig 3A). In part, this could be attributed to the significantly smaller size of maize starch grains recovered at this height when compared to those of cowpea (Chi squared = 15.18, p = 0.01).

#### Relationship between sampling position and DNA yield

Comparison of aerosol samples taken above the single-species bins revealed that maize consistently yielded more DNA than cowpea across all sampling positions (both heights and distances) and there was a progressive decline in DNA yield related to height for both species but less so for lateral distance (Fig 3B and 3C). Interestingly, aerosol samples at 60cm yielded roughly nine times as much aerosol DNA above maize grains than was recovered above cowpea at the same height. Microscopic examination of samples taken at this elevation revealed tissue fragments were present at roughly equal quantities for each species but maize starch particles were six times more concentrated than cowpea starch particles. This suggests that most of the DNA recovered from the aerosol samples originated from the starch particles rather than from the tissue fragments. This tenet is supported by the significant positive linear relationship that exists between DNA yield and abundance of starch particles along the vertical transect for both species (maize R^2^ = 0.751, p< = 0.026; cowpea R^2^ = 0.961, p< = 0.001).

### Sensitivity of aerosol sampling

The sensitivity of aerosol sampling was tested using the airborne ‘seed dust’ samples collected with the Coriolis sampler at 60cm height. This position was selected because of the relative stability in abundance of the starch particles across all replicates and the notable divergent ratio of the two particle types at this height (Fig 3A). Whilst there was some divergence between pure maize and pure cowpea HRM profiles generated by these samples, it was not possible to differentiate between individual samples within the titration series (S1 Fig).

**Fig 4:**
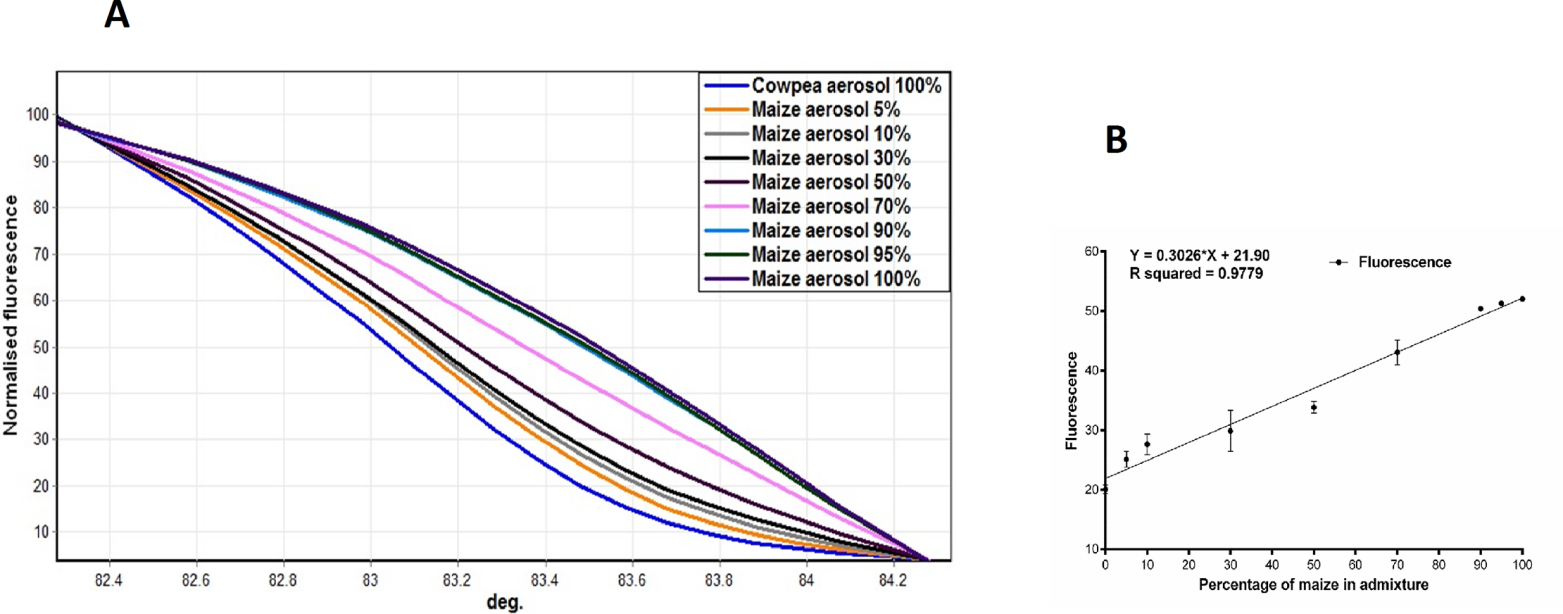
HRM profiles from aerosol samples above a titration of maize + cowpea seed mixtures. High Resolution Melting (HRM) analysis of *rbcL* amplicons generated from template DNA extractions recovered from aerosol samples collected 10cm above 10kg grain bins containing 0%, 5%, 10%, 30%, 50%, 70%, 90%, 95% and 100% of maize grains with cowpea grains. (**A**)Normalised HRM curves of aerosol samples collected above each part of the grain mixture titration series. (**B)** Linear regression for normalised HRM profile of all aerosol samples collected 10cm height above grain bins at a melt temperature of 83.5^°^C. Error bars represent standard error of the mean.

We next attempted to enhance signal strength by increasing the volume of air used to generate the sample (using a more powerful hand-held vacuum device) and by lowering the height of collection to 10cm. This strategy restored the ability of HRM analysis to differentiate between seed mixes across the titration (Fig 4A) and revealed a linear relationship between the HRM fluorescence and the percentage composition of the admixtures (Fig 4B). However, it appeared that low-level admixture (5–10%) of maize in cowpea was slightly more easily differentiated in the aerosol using HRM than low level admixtures of cowpea in maize.

### Mini barcode analysis by NGS

Next Generation Sequencing (NGS) analysis of admixture samples using the MiSeq platform was used to overcome the sensitivity limits exhibited by the HRM analysis. The 130 admixture samples sequenced on the MiSeq platform generated 15.05 million sequences that passed filter and were correctly assigned as *rbcL* sequences, of which maize comprised 49.3% and cowpea 50.7%, thus approximating the 1:1 ratio expected across all samples.

**Fig 5:**
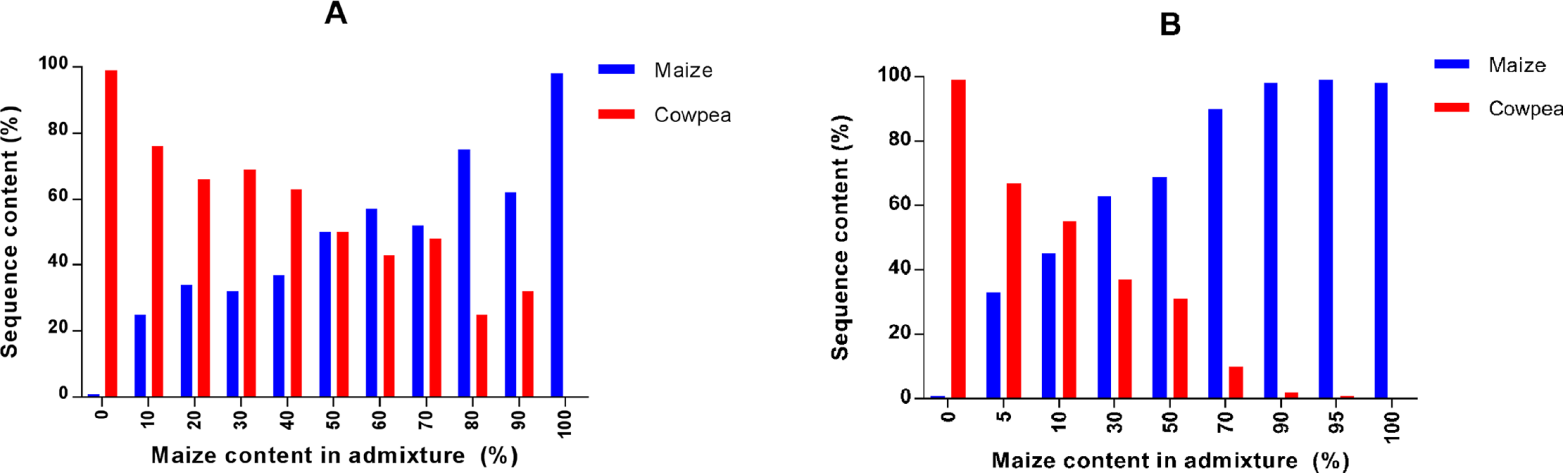
Species-specific minibarcode frequencies from aerosol samples collected above a titration of maize + cowpea seed mixtures. Frequency of maize and cowpea-specific mini-barcode sequences recovered from aerosol samples collected above individual grain bins in a titration series containing 0%, 10%, 20%, 30%, 40%, 50%, 60%, 70%, 80%, 90% and 100% (Coriolis samples) and 0%, 5%, 10%, 30%, 50%, 70%, 90%, 95% and 100% (vacuum samples) of maize grains in cowpea grains. (**A)** Histogram of the relative percentage of maize and cowpea mini-barcode sequences recovered from aerosol samples collected using a Coriolis aerosol sampler 60cm above bins representing the titration series of grain mixes. (**B)** Histogram of the relative percentage of maize and cowpea mini-barcode sequences recovered from aerosol samples collected using a hand-held, high power vacuum sampler 10cm above bins representing the titration series of grain mixes.

The sequence data recovered from aerosol samples collected on the Coriolis at 60cm height, showed that the proportion of maize sequence counts was broadly congruent with the order of increasing content of maize within the template mix (Fig 5A). The seed titration series constructed at 10cm height using the vacuum device was better able to detect low levels of maize in cowpea than cowpea in maize. However, despite this slight bias this analysis provided a more consistent and robust measure of the titration composition (Fig 5B).

## Discussion

The global commercial seed market was valued at US$42Bn in 2011, with internationally traded seed estimated to be US$8Bn in 2010 [39]. The huge volumes of seeds involved in international trade and the complexity of handling processes have led to numerous reports of unwanted seeds throughout the supply chain [3, 4]. Seed admixtures can occur in various forms such as unapproved GM seeds in approved GM seed lots, GM and non-GM seed admixtures, weed seeds and seeds of other crops in conventional seed lots [3, 7]. However, the conventional sampling approach used to detect the level of contamination involves direct collection of seed samples from the seed lot and has been reported to be inadequate for detecting patches of contaminants in seed lots [11, 13, 40]. The use of seed dust aerosol sampling to detect plant admixtures has potential to address the limitations of traditional seed sub-sampling. To date, however, the only reports of aerosol sampling have been for plant pollen analyses [22, 23, 28, 41]. Seed dust aerosol is created during handling (which involves operations such as loading, unloading, packaging, transport and storage) and processing of seeds. The focus of this study was therefore to determine the broad feasibility of using seed dust aerosol sampling to reliably detect and quantify contaminant seeds in seed lots.

HRM is a low-cost, rapid DNA analysis method that could be applied as a post-PCR assay on DNA extracted from aerosol samples such as those presented by seed dust. Previous researchers have applied the technique to study admixtures in a range of different contexts that include mutant screening [36, 42], detecting variety adulterations [43] and authenticity testing [44]. HRM measures the amount of fluorescence given off during DNA denaturation as the temperature is increased, and this is represented by the melting curve profiles generated [45]. The HRM assays in this study targeted amplicons generated within the *rbcL* gene using primers designed to be an exact match for both species used in the study (maize and cowpea) to minimise amplification bias which can occur when using universal primers [46]. Furthermore, the *rbcL* region was chosen for this study because although highly conserved, it contains enough informative Single Nucleotide Polymorphisms (SNPs) to discriminate between species and has been successfully used to identify taxa within metagenomic samples [33, 47]. Separation of the amplicons generated by the two species was possible by direct comparison of the HRM profiles (Fig 1A). Furthermore, the serial reflection of the different composition of the DNA mixes enabled the construction of a linear calibration relationship with an R^2^ value of 0.9889. This finding supports the findings of previous reports of barcode-HRM analysis being able to sensitively detect and quantify the components of admixtures [48–50]. In the present study, we found a clear separation of the various DNA mix combinations included in all titrations. This in turn showed a strong linear relationship between percentage DNA composition in the admixture and fluorescence at a given diagnostic temperature suggesting a high potential for use on a biological admixture (such as processed flour). However, when the same approach was applied to flour mixtures, HRM profiles were no longer capable of distinguishing between mixes containing 0–50% maize, although they were still able to separate higher proportions of maize, whereupon the linear relationship was retained. The simplest explanation of these results is that the two species of grain differ in the efficacy with which DNA can be extracted, with yields from maize being much lower than from cowpea such that insufficient amounts of maize DNA were recovered to allow their detection by HRM up to a threshold of 50%. Several researchers have reported that DNA extraction from seeds and subsequent amplification can be inhibited by polysaccharides, mucilage, lignin and other secondary metabolites [51–53] and that amount and composition of these molecules differ between species. Likewise, the composition of the cell walls of cereal grains are known to differ markedly from those in seeds of other higher plants, including dicotyledons [54–56], with potential to impact on efficacy of DNA extraction. It is also plausible that the recovery of plastid DNA (the template for *rbcL* amplification) was higher from cowpea flour than from maize. It is equally likely that cowpea yields more DNA extracted per grain of flour than maize, or that cowpea starch grains (amyloplasts) contain more plastid genome copies each than those of maize amyloplasts or simply that cowpea amyloplasts were more numerous per gram of flour. Whatever the cause, if such skewing is consistent across dilutions, its importance for the characterisation of seed admixtures depends heavily on the sensitivity of the method of detection deployed. For HRM analysis on flour samples, it was only possible to reliably detect the presence of maize when its presence exceeded 50% of the inter-species flour mix.

The characterisation of aerosol samples containing two species presents a greater challenge because bias caused by differential ease of DNA extraction can be multiplied by differences in the physical properties of the aerosol mix generated by each species. Here, we report that it was not possible to characterise the admixtures from aerosol samples at 60cm height suggesting that the levels of DNA recovered were too low and variable to allow for robust estimates of species composition at this height. However, there was a dramatic improvement in resolution when the volume of air sampled was greatly increased (through the increased airflow of the vacuum sampler) and raised dust concentration increased by lowering the sampling height to 10cm. Closer examination of the two main aerosol components arising from the grain bins (starch grains and tissue fragments) showed variation between the two species and this may help explain the observed discrepancy. Both maize and cowpea shared the same density of tissue fragments at 60cm above the bins whereas the airborne starch particles of maize were significantly more abundant than those of cowpea. Furthermore, a strong linear correlation was observed between starch grain abundance and DNA yields recovered from the aerosol samples (S1 Table). Considered together with the fact that the starch particles originate from amyloplasts and that the *rbcL* marker used in the study originates from the plastid genome, suggests the loss of signal at 60cm derives from the relatively high number of maize starch grains. Support for this premise can be taken from previous studies of starch particles in seeds of the two species. Mean size of starch particles from maize grains of four varieties ranged between 12μm and 34μm [57] but exhibited a trimodal size distribution, with around 5% of grains with a diameter of around 2μm seen in all genotypes. By comparison, mean starch particle diameter from four cowpea varieties ranged between 7.9μm and 15.5μm, and exhibited a simple unimodal variable size distribution [58]. Variability around the mean was low and so with nearly all particles exceeded 7 μm in all genotypes [58]. Thus, a high abundance of the smallest category of maize starch particles (2 μm) in aerosols above mixed seed batches offers the most plausible explanation for the disproportionately high recovery of *rbcL* maize sequences in such samples, and also the near absence of cowpea sequences above batches with low cowpea representation.

Clearly, the quantity of dust emanating from a 10kg bin of mixed seeds represents only a tiny fraction of that expected from a seed store or container ship. The extremely small size of the seed batches used here was intended to allow for particulate sedimentation between replicate sampling events and so minimised carry-over effects. Even so, several hours were still required to ensure aerosol seed particles had cleared from the room atmosphere before renewed sampling took place. Conversely, the disadvantage of using a small seed source is that it risks reducing the amount of extractable DNA in the aerosol falling below detectable levels for at least one or more of the component species. This limitation proved to be the case when using the Coriolis samples collected at 60cm, where the relationship between titration composition in the seed mix and the associated aerosol HRM profile was lost. However, by taking the simple measures of reducing altitudinal height to 10cm where particulate density was higher and by increasing the volume of air sampled, this relationship was restored (Fig 4B). Thus, density of extractable DNA in the aerosol appears to be a key limiting factor in determining the usefulness of HRM as a low-cost option for seed lot characterisation. Consideration should also be given to the effect of seed morphology and physiology on the nature of the aerosols created. Like many cereals, grains of maize and cowpea are of roughly comparable size and structure, and contain copious quantities of starch-rich amyloplasts. There were nevertheless clear physical differences seen here in the physical composition of the aerosols generated by the two species. Such differences have potential to introduce bias when using aerosols to quantitatively reconstruct the species composition of the seed source, and require careful characterisation to a reference seed source titration (as here) before the aerosol profiles can be usefully interpreted. However, there are some testing scenarios where the characteristics of the two seed types of interest will be identical and so do not require this form of correction. For instance, aerosols above mixtures of GM and non-GM grain shipments, or above mixtures of industrial and food seed types (e.g. as in oilseed rape, *Brassica napus*) should produce identical aerosol profiles for each seed type. In such situations, there should be no DNA extraction bias between seed types and so the relationship between source mixture and HRM signal should more closely match observed DNA titration relationships. Even then, further development would still be required before aerosol sampling could be used as a quantitative test for seed admixture. The detection of unauthorised GM admixtures requires a comprehensive collection of event- and construct-specific primers, and these would need to be compared against negative control primers to cover a range of admixture possibilities [59]. While the present work may be taken to imply that sensitivity of aerosol detection by either HRM or Next Generation Sequencing would be sufficient at least for the detection of current importation thresholds of 5% into the USA and Canada [9], consideration is required of other contributing factors. The first of these relates to our use of the plastid marker (*rbcL*). Plastid markers such as this have a high copy number per cell and so increase the sensitivity of target DNA detection from aerosol samples, especially here because of the relatively high abundance of amyloplasts in the aerosol. However, this choice of marker also means that our findings have direct relevance only to the few examples of transplastomic GM events [60] rather than to the majority of commercial GM events in which the transgene is integrated into the nuclear genome [61]. For an aerosol-based system of seed mixture be developed for this context, the target nuclear DNA would need to originate from tissue fragments observed in the aerosol rather than from the amyloplasts (which lack nuclear DNA). Given the lower density of these fragments and the far lower abundance of the target primer binding sites within each cell they contain, it is likely that detection of GM admixtures would require aerosols with much higher particulate densities. There are nevertheless grounds to argue that this would be a reasonable expectation in most commercial settings. In the present study, we were able to detect and quantify species identity at 5% from 10 kg seed bins. This means that the aerosol template originated from just 500g of source seed. Grain elevators can hold several million kg of grain [62] and aerosols above them can contain as much as 250 mg/m^3^ of airborne seed particulates [63]; sufficient to cause long-term respiratory problems for associated workers [18]. Given that particle pellets collected in the present work originated from about 600ml of air invariably and yielded <5μl of particulates (= 5μg = 5 /0.6 = 8.3 μg/ l), it follows that airborne densities above commercial grain stores are likely to be at least tens of thousand times higher than those observed here. Under these circumstances, template abundance is very likely to be sufficient to allow detection of even low-level presence of nuclear markers. In any event, given the capability to detect 5% admixture under the current, minimal density aerosol environment, the exceptionally high particle densities associated with grain stores opens up the possibility aerosol sampling should be able to detect trace levels of particle admixture using plastid or mitochondrial barcode markers. There are numerous scenarios in the passage of traded seed lots where this capability could offer tangible benefits to the industry. For example, for sufferers of coeliac disease, livelong avoidance of wheat, barley and rye represents the only effective management of the disease [64]. Oats and oat products often form an important part of the diet of coeliac patients although inadvertent admixtures during the supply chain can cause health issues and coincidentally reduce value of the products. A recent study of oat grain samples and oat products found that 109 of 134 contained detectable but unquantified amounts of wheat, barley and rye [65]. The development and application of an aerosol-based test for admixture throughout the supply line could help manage this problem. Similarly, organelle barcode markers Cytochrome Oxidase I (*COI*) and maturase K (*matK*) could potentially be used in a similar fashion to *rbcL* here, to detect pests (from air-borne tissue and faecal templates), weed seeds or of invasive plants or animals.

The use of HRM as a means of characterising DNA mixtures recovered from aerosol samples offers the advantage of speed and low infrastructure set-up costs. On the other hand, the absence of sequence information means that the approach is susceptible to potential misdiagnosis and would be unable to accommodate for previously uncharacterised materials (such as a new pest species or unexpected seed admixture). The application of a metabarcoding approach using the Illumina MiSeq platform provided an effective strategy to overcome the limitation of HRM to detect differences arising from small quantities of template. Other studies have used metabarcoding to assess components of DNA mixtures [66–68], especially to reconstruct diets from faecal samples [69–71]. Key limitations of using next-generation DNA sequencing such as on the Illumina MiSeq platform for routine screenings of eDNA samples include the cost and time involved to generate the results and the infrastructure required. While the use of dual labelled indexing in the library construction stage considerably reduced the unit cost of analysis in our study, the time required to generate and analyse results currently precludes this strategy from many commercial settings. However, the recent development of the Nanopore portable sequencing platform (Oxford Nanopore Technologies, UK) may be able to address these limitations as shown by the ability to sequence entire genomes within hours using an ‘al fresco’ laboratory [72].

The choice of an appropriate DNA marker system clearly represents an issue that requires careful thought. Here, we used custom-designed primers for the *rbcL* locus because the identity of species involved was known *a priori*. In scenarios where the emphasis is on detecting diverse, unknown species (such as the screening for pests, invasive plant seeds or diseases), it is most likely that conventional barcoding loci and primers would need to be utilised. However, variability between species in the binding efficiency of universal barcoding primers [66, 73] represents a significant problem for the quantification of the species components of aerosol samples. Moreover, species with imperfect primer binding run the risk of being absent or grossly underrepresented. In the present study, we ensured that the *rbcL* primers used were perfect matches for both species under study. This simple precaution becomes more challenging as the number of potential species expands but could be adopted to assess the finite number of seed crops (and major weeds) expected in major shipments. An alternative approach for shipments likely to contain unknown samples would be to use smaller and more reliably amplified barcoding markers such *ndhJ*, *ndhK*, *rpoB* and *rpoC1*. While these markers yield fewer sequences differences between sister species, they should nevertheless be able to differentiate crop seeds from those of weeds. Furthermore, since their primer binding sites are less variable, amplification is likely to be more consistent across wide taxonomic groups [74].

## Conclusions

Results from our study show that the efficacy of DNA extraction from grains vary with species, and this variance may be attributed to the different composition of seed species. Furthermore, the sensitivity of the detection method adopted is critical in characterising seed admixtures. Our study has illustrated that there is great potential for using aerosol samples to quantify the species composition of seed mixtures. However, the practical utility of the approach will ultimately depend on considerable further refinement of the method and its adaptation for use on large-scale seed storage and transport facilities.

## Supporting information

S1 Fig Variation in *rbcL* melting profiles from aerosols above maize-cowpea grain mixes. Scatter plot showing the mean fluorescence values of a normalised HRM profile at 83.2^°^C generated by *rbcL* amplicons recovered from aerosol samples collected 60cm above bins containing a titration of maize and cowpea grain mixes using a Coriolis sampler. The bins contained one of the following mixtures of maize grains (with residual cowpea grains): 0%, 10%, 20%, 30%, 40%, 50%, 60%, 70%, 80%, 90%, and 100%. The figure shows that the capacity of HRM to distinguish between the different mix compositions at 60cm height was lost. Error bars indicate standard error of the mean.
(PDF)

S1 Table Starch particle and DNA yields above grain bins of maize and cowpea. Correlation analysis between abundance of starch particles and DNA yield of maize and cowpea aerosol samples collected at different sampling heights (5, 10, 15, 30, 60 and 120cm). The table shows a strong linear correlation between abundance of starch granules and DNA isolated from the aerosol samples.
(PDF)
