# Supplementary material for: The potential of aerosol eDNA sampling for the characterisation of commercial seed lots
Source: PLoS One. 2018 Aug 1;13(8):e0201617. doi: 10.1371/journal.pone.0201617 (PMC6070268; doi:10.1371/journal.pone.0201617)

S1 Fig. Variation in *rbcL* melting profiles from aerosols above maize-cowpea grain mixes. Scatter plot showing the mean fluorescence values of a normalised HRM profile at 83.2°C generated by *rbcL* amplicons recovered from aerosol samples collected 60cm above bins containing a titration of maize and cowpea grain mixes using a Coriolis sampler. The bins contained one of the following mixtures of maize grains (with residual cowpea grains): 0%, 10%, 20%, 30%, 40%, 50%, 60%, 70%, 80%, 90%, and 100%. The figure shows that the capacity of HRM to distinguish between the different mix compositions at 60cm height was lost. Error bars indicate standard error of the mean.

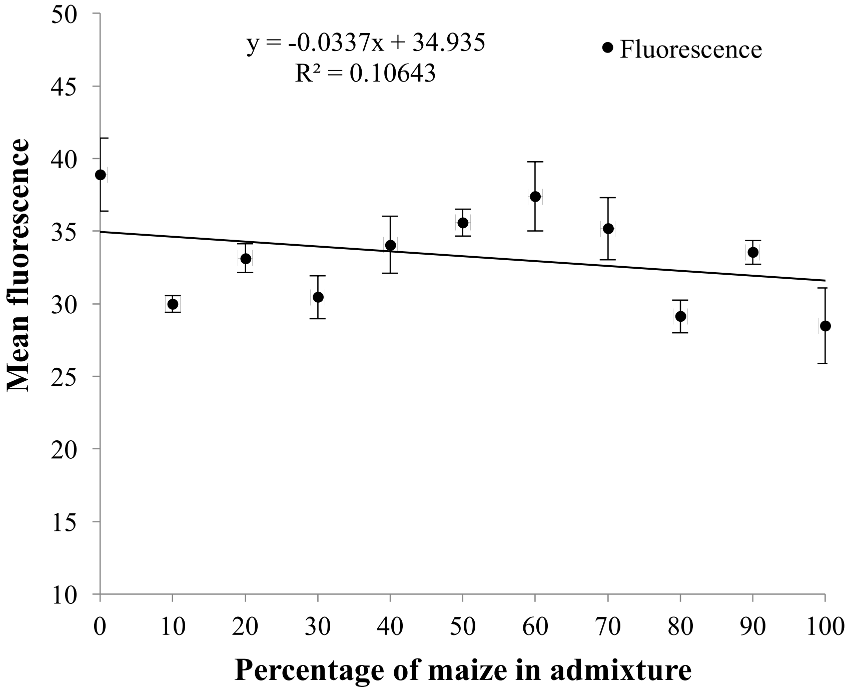

Supplement: S1 Fig — Scatter plot showing the mean fluorescence values of a normalised HRM profile at 83.2°C generated by rbcL amplicons recovered from aerosol samples collected 60cm above bins containing a titration of maize and cowpea grain mixes using a Coriolis sampler. The bins contained one of the following mixtures of maize grains (with residual cowpea grains): 0%, 10%, 20%, 30%, 40%, 50%, 60%, 70%, 80%, 90%, and 100%. The figure shows that the capacity of HRM to distinguish between the different mix compositions at 60cm height was lost. Error bars indicate standard error of the mean. (PDF) [file pone.0201617.s001.pdf]
