# Supplementary material for: The potential of aerosol eDNA sampling for the characterisation of commercial seed lots
Source: PLoS One. 2018 Aug 1;13(8):e0201617. doi: 10.1371/journal.pone.0201617 (PMC6070268; doi:10.1371/journal.pone.0201617)
Supplement: S1 Table — Correlation analysis between abundance of starch particles and DNA yield of maize and cowpea aerosol samples collected at different sampling heights (5, 10, 15, 30, 60 and 120cm). The table shows a strong linear correlation between abundance of starch granules and DNA isolated from the aerosol samples. (PDF) [file pone.0201617.s002.pdf]

S1 Table. Starch particle and DNA yields above grain bins of maize and cowpea. Correlation analysis between abundance of starch particles and DNA yield of maize and cowpea aerosol samples collected at different sampling heights (5, 10, 15, 30, 60 and 120cm). The table shows a strong linear correlation between abundance of starch granules and DNA isolated from the aerosol samples.

	r	p-value	R^2
Maize	0.866	*0.026	0.751
Cowpea	0.980	*0.001	0.961

*p-values are significant at a significance level of 0.05
